# Bile Acid-Induced Arrhythmia Is Mediated by Muscarinic M_2_ Receptors in Neonatal Rat Cardiomyocytes

**DOI:** 10.1371/journal.pone.0009689

**Published:** 2010-03-15

**Authors:** Siti H. Sheikh Abdul Kadir, Michele Miragoli, Shadi Abu-Hayyeh, Alexey V. Moshkov, Qilian Xie, Verena Keitel, Viacheslav O. Nikolaev, Catherine Williamson, Julia Gorelik

**Affiliations:** 1 National Heart and Lung Institute, Imperial College London, London, United Kingdom; 2 Institute of Reproductive and Developmental Biology, Imperial College London, Hammersmith Campus, London, United Kingdom; 3 Clinic of Gastroenterology, Hepatology and Infectiology, Heinrich-Heine-University, Düsseldorf, Germany; 4 Faculty of Medicine, Universiti Teknologi MARA, Shah Alam, Selangor, Malaysia; 5 Heart Centre, First Hospital of Hebei Medical University, Shijiazhuang, Hebei Province, China; Instituto de Química, Universidade de São Paulo, Brazil

## Abstract

**Background:**

Intrahepatic cholestasis of pregnancy (ICP) is a common disease affecting up to 5% of pregnancies and which can cause fetal arrhythmia and sudden intrauterine death. We previously demonstrated that bile acid taurocholate (TC), which is raised in the bloodstream of ICP, can acutely alter the rate and rhythm of contraction and induce abnormal calcium destabilization in cultured neonatal rat cardiomyocytes (NRCM). Apart from their hepatic functions bile acids are ubiquitous signalling molecules with diverse systemic effects mediated by either the nuclear receptor FXR or by a recently discovered G-protein coupled receptor TGR5. We aim to investigate the mechanism of bile-acid induced arrhythmogenic effects in an *in-vitro* model of the fetal heart.

**Methods and Results:**

Levels of bile acid transporters and nuclear receptor FXR were studied by quantitative real time PCR, western blot and immunostaining, which showed low levels of expression. We did not observe functional involvement of the canonical receptors FXR and TGR5. Instead, we found that TC binds to the muscarinic M_2_ receptor in NRCM and serves as a partial agonist of this receptor in terms of inhibitory effect on intracellular cAMP and negative chronotropic response. Pharmacological inhibition and siRNA-knockdown of the M_2_ receptor completely abolished the negative effect of TC on contraction, calcium transient amplitude and synchronisation in NRCM clusters.

**Conclusion:**

We conclude that in NRCM the TC-induced arrhythmia is mediated by the partial agonism at the M_2_ receptor. This mechanism might serve as a promising new therapeutic target for fetal arrhythmia.

## Introduction

Intrahepatic cholestasis of pregnancy (ICP) is a maternal metabolical disease characterised by raised maternal serum bile acids. It can be complicated by fetal distress, intrauterine death, and pre-term labour [1]–[4]. ICP is common, affecting 1 in 200 pregnancies in the UK [5]and is more prevalent in other countries, e.g. Chile where it affects up to 5% of pregnant women [6]. The aetiology of the fetal death is poorly understood. It is thought to occur suddenly, as there is no evidence of preceding utero-placental insufficiency and the fetal autopsy is normal[1]. Bradycardia and an abnormal fetal heart rate (≤100 or ≥180 beats/minute) have been observed in some studies with ICP [7], [8]. Furthermore there was a case report of fetal tachyarrhythmia in association with atrial flutter during labour in ICP [9]. Tauro-conjugated primary bile acids predominate in cholestatic pregnancies, and our previous work has focussed on the influence of a tauro-conjugate of cholic acid (TC) on cardiomyocyte function[10]. Previously, we hypothesised that raised fetal serum bile acids in ICP cause fetal arrhythmia and sudden intrauterine death and demonstrated that TC can acutely alter the rate and rhythm of cardiomyocyte contraction and cause abnormal Ca^2+^ dynamics of single cells[11]. Superfusion of a culture with TC had the arrhythmogenic effects in individual cells, such as a reduction in amplitude of contraction as well as Ca^2+^ related arrhythmias [11], [12]. However, the molecular mechanisms of bile-acid induced arrhythmogenesis are still elusive.

There is growing evidence that bile acids are versatile signalling molecules endowed with systemic endocrine functions[13]. It is likely that transport of bile acids across the plasma membrane can influence their adverse effects on contraction and calcium dynamics. The repertoire and properties of fetal transporter proteins may differ from those in the adult and this may explain the more potent effect of bile acids on fetal cardiomyocytes. In hepatocytes, bile acids are tightly regulated due to their inherent toxicity[14], [15]. Transported into the cell, bile acids modulate several nuclear hormone receptors including farnesoid X receptor (FXR; also known as NR1H4) which regulate downstream functions in a genomic signaling manner[16]–[21]. While the pathways of bile homeostasis are becoming increasingly characterised in hepatocytes, little is known about pathways that are involved in bile acid effects on cardiomyocytes. Our previous data have confirmed that the genes encoding FXR, BSEP and MDR3 are expressed in human cardiomyocyte cultures[22], adult rat hearts, fetal rat hearts and neonatal rat cardiomyocyte cultures.

Recently, a bile acid-specific G-protein-coupled receptor TGR5 (also known as GPBAR1, M-BAR and BG37) has been identified[23]. This receptor has been associated with immunomodulatory properties of bile acids[23] and some hepatic functions[24]. In endothelial cells TGR5 regulates nitric oxide production via cyclic AMP-dependent activation of endothelial nitric oxide synthase[25]. However, the presence of TGR5 in cardiomyocytes has not been investigated. Some other reports have suggested that bile acids might affect muscarinic cholinergic receptor signaling[26]. Stimulation of muscarinic receptors on colon cancer cells by bile acids was attributed to cell proliferation and neoplasia[26]. **The bile acid TLCA has been shown to interact with M_3_ isoform of muscarinic receptor in chinese hamster ovary (CHO) cells expressing rat or human muscarinic receptor** **[27]** **.** In some other cell types such as gastrointestinal smooth muscle cells, biliary epithelium, vascular endothelium and dermal neurons bile acids have been suggested to interact with muscarinic receptors. However, little is known about the possible action of bile acids on cardiac muscarinic receptors[26].

Here we extensively investigated the mechanism of bile-acid induced arrhythmogenesis and found that TC is a partial agonist of the muscarinic M_2_ receptor and that its arrhythmogenic effect in fetal cardiomyocytes is mediated via inhibition of cAMP and impairment of contraction.

## Results

### Fxr and TGR5 are not involved in TC-induced arrhythmia

**Figure 1:**
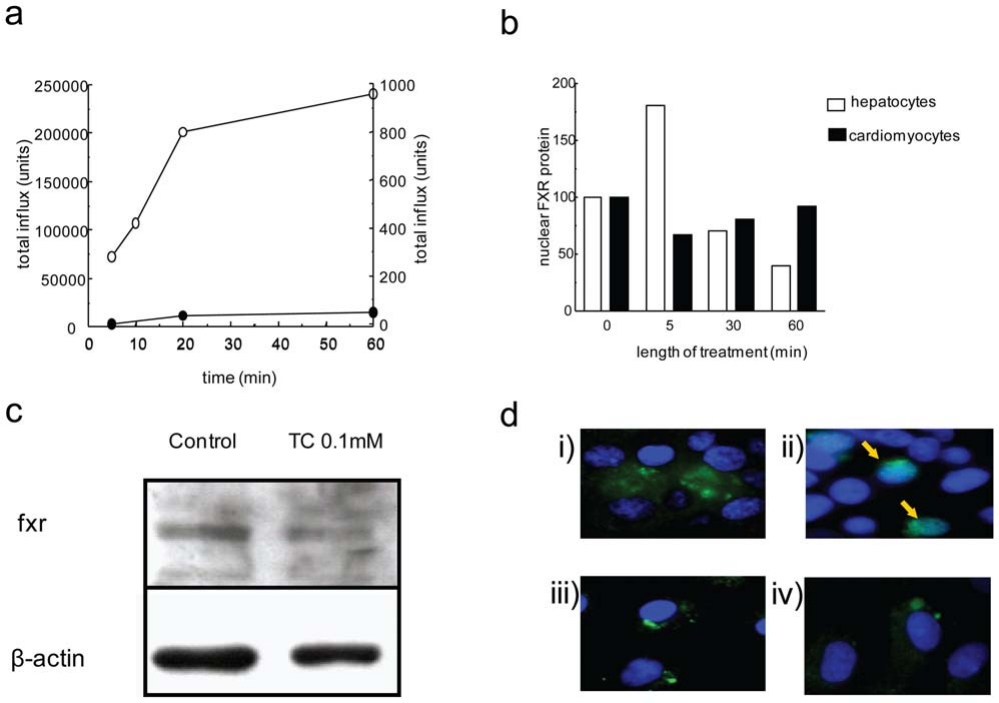
Fxr interaction with TC in NRCM. (a) The 3H TC influx assay showed low uptake of 3H TC in NRCM (closed circle) compared to primary human hepatocytes (open circle). While hepatocytes showed uptake of 200000 units of TC in the first 20 minutes, cardiomyocytes did not take up more than 50 units of radioactive TC. fxr in neonatal cardiomyocytes (b-d). (b) Graph quantifying the relative expression of fxr protein from western blots performed using NRCM and the human hepatocyte cell line Huh7 treated with TC 0.1 mmol/L at 0, 5, 30 and 60 minutes. (c) Representative western blot showing expression of fxr protein in rat neonatal cardiomyocytes with and without addition of TC. (d) Control human hepatocyte cell line Huh7 stained for fxr; pre (i) and post-bile acid treatment (ii). NRCM stained for fxr; control (iii) and after 1 incubation with TC 1 mmol/L (iv). FITC (green) = fxr; DAPI (blue) =  nucleus; yellow arrow fxr nuclear region staining. (n = 3 observations).

**Figure 2:**
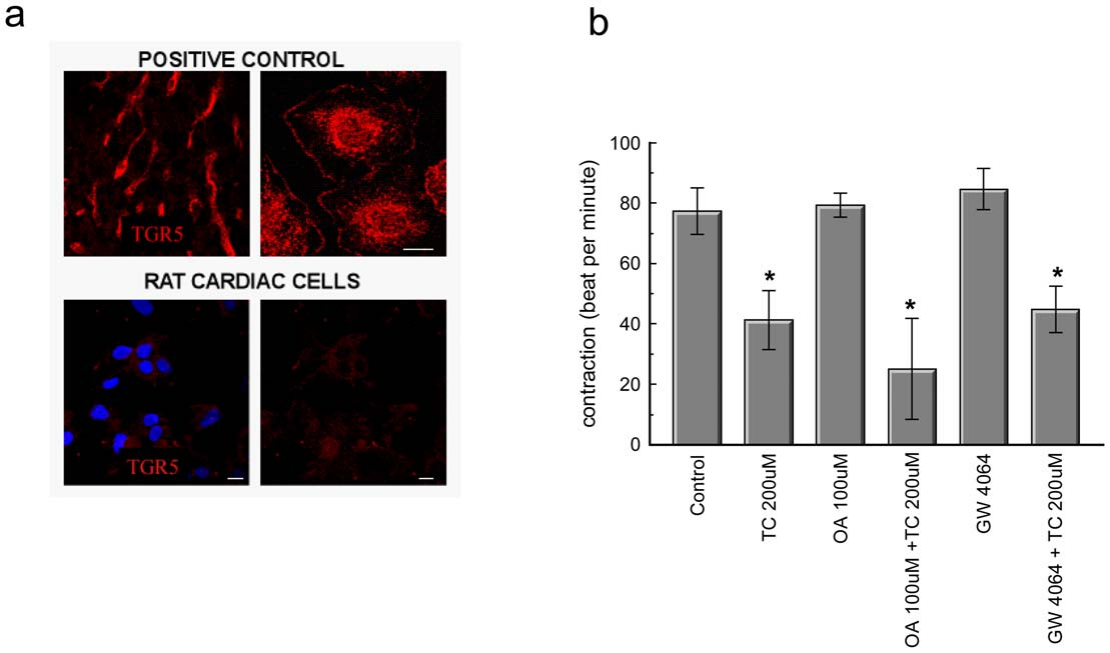
Involvement of TGR5 and fxr in TC induced arrhythmia in NRCM. (a) Lack of expression of TGR5 in cardiac cells. Top panels: TGR5 immunolocalisation in a **rat** liver section (left) and cultured **rat** Kupffer cells (right) Scale bar 10 μm. Bottom panels. Immunostaining for TGR5 in a cluster of NRCM (left and right panels, red colour). Nuclei are stained with Hoechst dye (left panel, blue colour) (b) Graph demonstrating that the TGR5 agonist Oleanolic acid (0.1 mmol/L) and the fxr agonist GW4064 (0.01 mmol/L) do not protect NRCM from TC-induced arrhythmia. (* Control vs P<0.05); n = 3 observations).

Initially we investigated whether the bile acids act through receptors on the cell surface and whether they are transported into the cytoplasm of NRCM where they interact with nuclear receptors. First we quantified the cellular uptake of radioactively labeled TC which appeared to be negligible in NRCM compared to primary human hepatocytes (Fig. 1a). We studied nuclear translocation of fxr, as a marker of fxr activation. We did not observe significant translocation of fxr in NRCM treated with TC as observed in human hepatocyte cell line Huh7 after 5 minutes treatment with TC (Fig. 1b). Therefore interaction with nuclear receptors was not considered to be of relevance to cardiac cells. Indeed, expression of bile transporters in cardiomyocytes was generally lower than in hepatocytes (Table S1) and, importantly, despite its abundant expression in cardiomyocytes (Fig. 1c), fxr did not show a typical redistribution from the cytoplasm to the nucleus upon treatment with bile acids (Fig. 1b,d). Likewise, we could not detect any expression of the bile acid specific TGR5 in NRCM by immunohystochemistry (Fig. 2a). In line with these findings, agonists of fxr (GW4064) and TGR5 (oleanolic acid) also did not influence cardiomyocyte contraction, nor did they protect against TC-induced arrhythmia (Fig. 2b).

### TC is a partial agonist at the muscarinic M_2_ receptor

**Figure 3:**
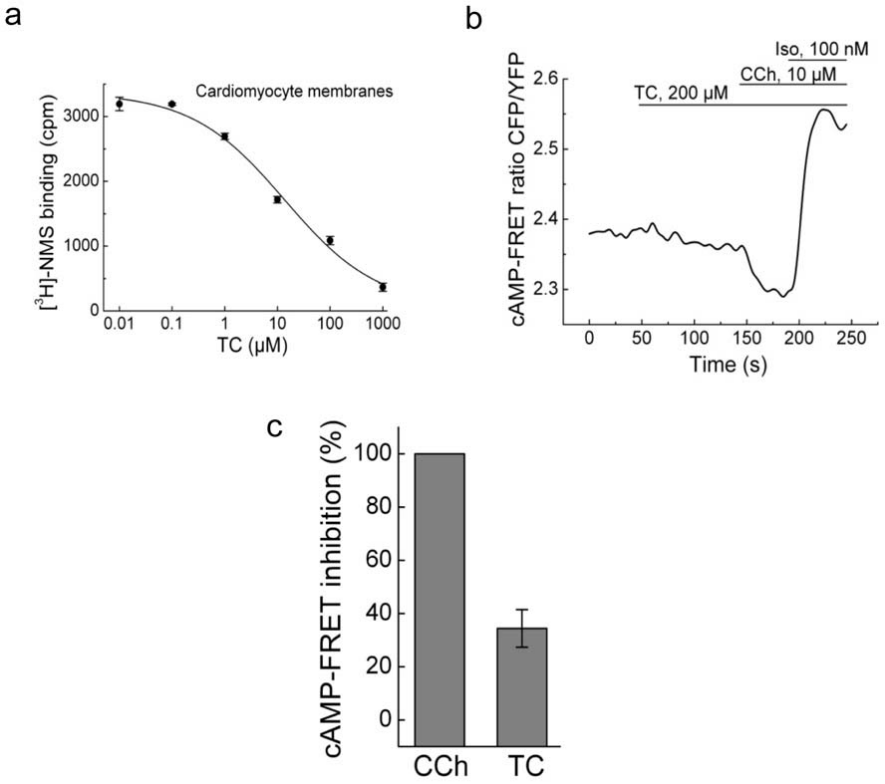
TC is a partial agonist of the M_2_ muscarinic receptor. (a) Specific binding of TC to muscarinic receptors in cardiomyocyte membranes. Competitive displacement of ^3^H-NMS with TC is shown (*K*_d_ values were 17.2±7.3 µmol/L, n = 3). (b) FRET-based cAMP measurements in NRCM upon stimulation with TC show its partial agonistic effect compared to the full agonist carbachol (CCh). Isoproterenol (Iso) was used a positive control to stimulate cAMP production. Representative experiment (n = 5), quantification is shown in (c).

Since the involvement of muscarinic receptors in bile acid signalling has been previously suggested in non-cardiac cells[26], we sought to investigate whether TC can bind to and activate the M_2_ receptor subtype, which is most relevant for cardiomyocyte physiology. First, we prepared cell membranes from cultured neonatal cardiomyocytes and performed radioligand binding assays with the muscarinic receptor antagonist N-methylscopolamine. Similar to our observations in muscarinic receptor-overexpressing CHO-M2 cells (Fig. S1), competitive binding displacement experiments showed that TC can bind to the muscarinic receptors endogenously expressed on cardiomyocytes with an apparent Kd of ∼17 µmol/L (Fig. 3a).

To study whether TC can act as a ligand of the M_2_ receptor and induce downstream signaling responses, we analyzed the inhibitory effect of TC on cAMP production in NRCM expressing the FRET-based cAMP sensor Epac2-camps[28]. Treatment of cells with TC led to a decrease in basal cAMP levels which reached its maximum after ∼1 min, similar to the full receptor agonist carbachol. However, the TC-induced inhibition was partial (∼30%) compared to the effect of carbachol (Fig. 3b, c), suggesting that TC acts as a partial agonist of this receptor.

### Muscarinic M_2_ receptor mediates TC effects of cardiac contraction

**Figure 4:**
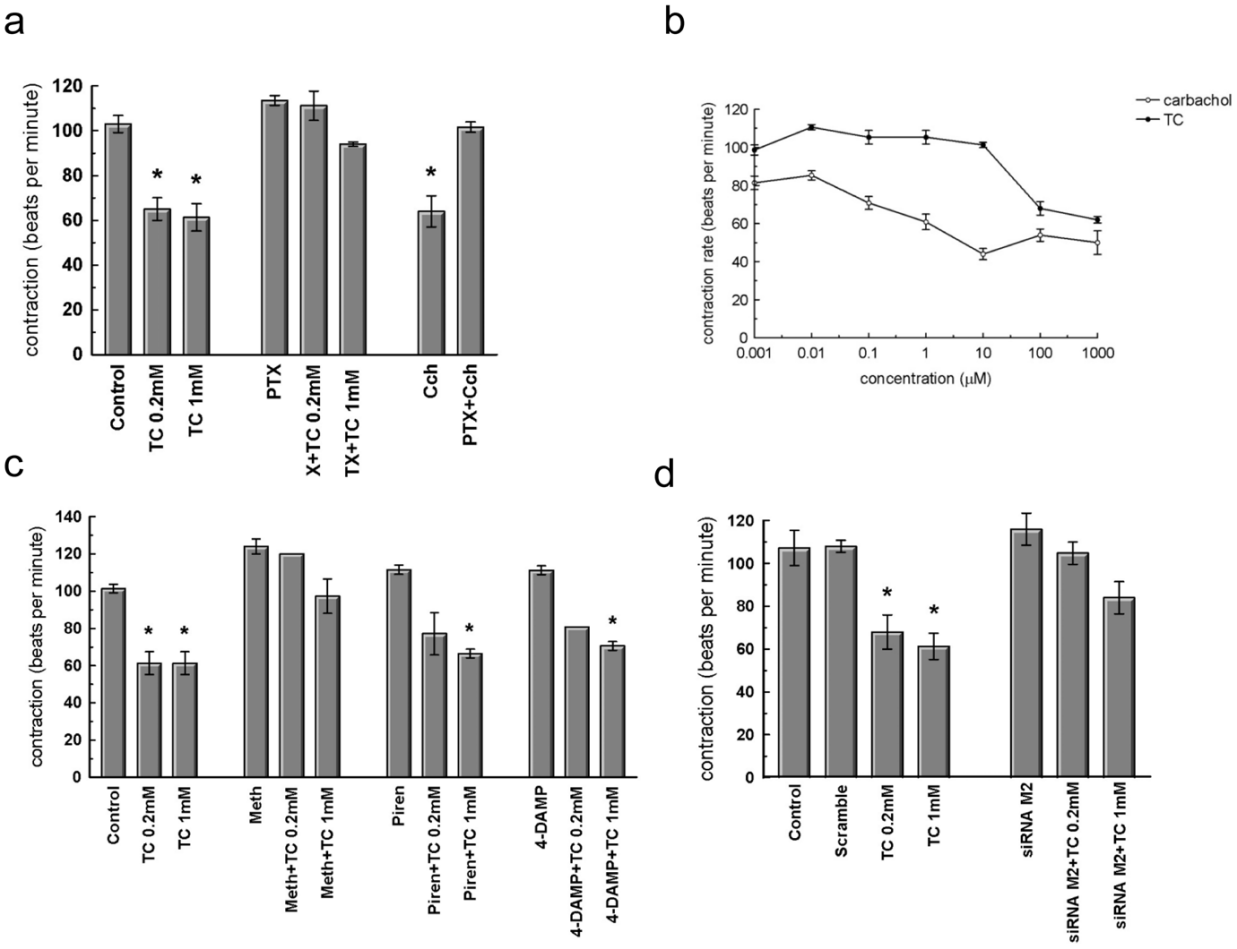
The muscarinic receptor is involved in TC-induced arrhythmia. Contraction of NRCM expressed as beat per minutes (bpm). (a) Treatment with TC or the muscarinic agonist carbachol in the presence or absence of the Gi protein blocker PTX. (b) Dose dependent effect of carbachol (CCh) and taurocholate (TC). (c) Pharmacological inhibition of muscarinic receptors, treatment with M_1_, M_2_ and M_3_ muscarinic receptor antagonists. (d) Scramble (non-targeting) siRNA and M_2_ siRNA knockdown of cells. (* Control vs P<0.001); n≥3 observations).

Prior to addition of TC the NRCM contracted spontaneously at a rate of about 106 bpm±6.08 (mean±SEM, n = 5). When TC 0.2 mmol/L and 1 mmol/L were added to the culture medium of these cells there was a significant reduction in the rate of contraction, 61.20 bpm±5.33 and 61.32 bpm±7.55 (mean±SD, n = 6. P<0.001) respectively (Fig. 4a, c). To investigate whether the inhibitory effect of TC on cardiomyocyte contraction is mediated by muscarinic receptors, we used pharmacological inhibition and knock-down approaches. Preincubation with either pertussis toxin to inhibit G_i_–proteins or with the M_2_ receptor-specific inhibitor methoctramine abolished the negative chronotropic effect of TC after 10 minutes treatment with either 0.2 or 1 mmol/L TC (Fig. 4a, c). The muscarinic receptor agonist, carbachol, caused a similar reduction in contractility to that observed with TC but with much higher potency (Fig. 4b). In contrast, preincubation with either M_1_ receptor-specific inhibitor pirenzepine or M_3_ receptor-specific inhibitor 4-diphenylacetoxy-N-methylpiperidinemethiodide did not inhibit the bile acid-induced reduction in contraction (Fig. 4c), suggesting that M_2_ receptors are responsible for the effect of TC on contraction. We further confirmed this observation by siRNA knockdown of the M_2_ receptor. Transfection of NRCM with siRNA led to a 65% reduction in the receptor mRNA expression (Fig. S3) and and substantially reduced the impact of high concentration of TC 1 mmol/L (Fig. 4d) and **0.1 mmol/L carbachol on the rates of contraction (Fig. S2).**

**Figure 5:**
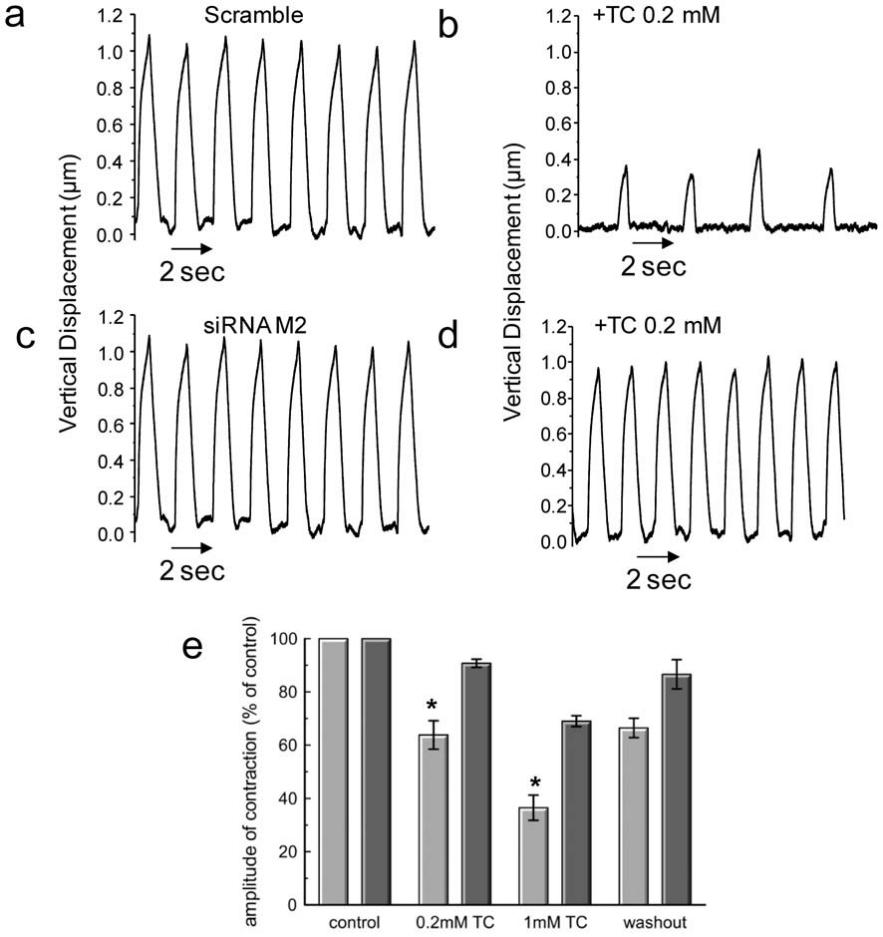
Muscarinic M_2_ receptor mediates TC effects of cardiac contraction. Representative measurement of the amplitude contraction of NRCM using SICM.(a-b) Scramble (non-targeting) siRNA NRCM showed regular contraction and alterations in rhythm and amplitude following TC treatment.(c-d) cardiomyocytes showed regular contraction following siRNA knockdown of M_2_ and TC treatment did not have an effect on rhythm of contraction. (e) Graph demonstrating the influence of adding 0.2 mmol/L and 1.0 mmol/L TC on the amplitude of contraction of non-targeting siRNA (grey bar) and siRNA-M_2_ knockdown (black bar) of NRCM. This is represented as a percentage (%) of the amplitude of contraction in cells prior to the addition of TC (designated controls). The extent to which the amplitude of contraction returns to normal after transfer of cells into TC-free medium is also shown. (* Control vs P<0.05); n = 6 observations).

The data obtained using optical contractility measurements were further substantiated by scanning ion conductance microscopy (SICM), a new advanced microscopic technique. Recordings of the vertical displacement of contractions by SICM revealed that TC not only reduced the frequency of contraction but also made cardiomyocyte contraction irregular (Fig. 5a,b,e, Fig. S4). However, this TC effect was abolished by M_2_ receptor knockdown (Fig. 5c, d, e), even at a TC concentration of 1 mmol/L. (Fig. S5).

### Functional involvement of the muscarinic M_2_ receptor in the TC-induced calcium desynchronization

**Figure 6:**
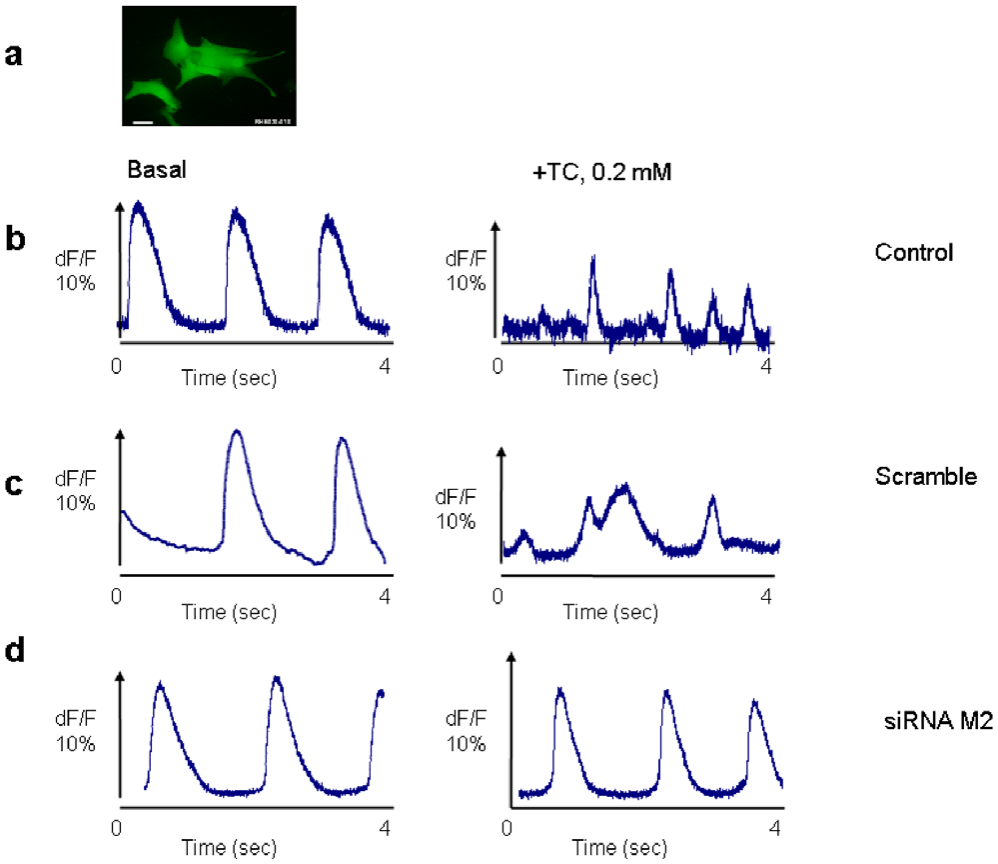
Functional involvement of the muscarinic M_2_ receptor in the TC-induced calcium desynchronization. (a) Overview of a cluster loaded with 5 µg/ml Fluo-4 AM (scale bar  = 10 µm). Representative of intracellular Calcium dynamics recorded in NRCM pre and post-treatment with 0.2 mmol/L TC. (b) Ca ^2+^ transients recorded in untreated (left panel) and treated (right panel) of control cells. (c) Ca ^2+^ transients recorded in untreated (left panel) and treated (right panel) of scramble (non-targeting) siRNA knockdown cells. (d) Ca ^2+^ transients recorded in untreated (left panel) and treated (right panel) of siRNA M_2_ knockdown cells. n>5 observations.

The effect of TC on calcium desynchronization and the involvement of M_2_ receptors in this process were investigated in small 3 days old NRCM clusters (∼100×100 µm, Fig. 6a, Video S1). Optical recordings of Ca^2+^ transient amplitude for 4 seconds before and after acute exposure (20 min) to 0.2 mmol/L TC showed a complete loss of Ca^2+^ transient synchronisation in control clusters and in clusters of cells transfected with scrambled siRNA clusters after TC treatment (Fig. 6b,c, Video S1). In contrast, cell clusters transfected with siRNA targeting the M_2_ receptor, did not show these abnormalities (Fig. 6d). The maximum Ca^2+^ peak in the presence of TC scaled to pre-treatment clusters showed a significant reduction in control and non-targeting siRNA knockdown (P<0.05) clusters, whereas the reduction in clusters with siRNA transfection was less marked and comparable with the control group (Fig. 6b-d, Table 1).

**Table 1:** Effects of 0.2 mmol/L TC on Ca^2+^ amplitude and percentage of desynchronized cluster after treatment with TC.

Condition of cells	Percentage of calcium peak reduction compared to pre-treatment (Means ± SEM)	Loss of Ca 2+ Transient Synchronization after TC (% of cells)
Control	45.4±11.35	67
Scrambled siRNA	38.7±3.87	70
siRNA M_2_	7.6±0.28	3.57

Data are normalized to pre treatment condition and scale accordingly to dF/F 10%, n>5 observations.

## Discussion

ICP is a severe disease of pregnancy associated with detrimental effects of bile acids on the fetal heart. The molecular mechanisms of this cardiotoxicity have remained elusive. To unravel the mechanism of the bile-acid induced arrhythmia, we first hypothesized that the effects of nuclear receptors activated by bile acids transported into cardiac cells might be involved. However, we have shown that all the main bile acid transporters are expressed at relatively low levels in the fetal heart and in neonatal cardiomyocyte cultures. Our data are consistent with previous published data [29].

Interestingly, the nuclear receptor FXR [30], [31] which regulates target genes that influence bile, glucose homeostasis and lipoprotein metabolism in the liver is highly expressed not only in bile acid-target organs, like liver and gut but also in the kidney, adrenal glands, thymus and heart[32]–[34]. It has been suggested that FXR ligands play roles in cardiovascular diseases[34], [35].

Surprisingly, although the bile acid nuclear receptor fxr is expressed in cardiomyocytes, it did not undergo nuclear translocation upon treatment with TC (Fig. 1). In line with the data on low expression levels of bile acid transporters in cardiomyocytes (Table S2), the uptake of radioactively labeled TC into these cells was negligible compared to human hepatocytes, and the fxr receptor agonist GW4064 had no effect on contraction.

This led us to hypothesise that TC acts on cardiomyocytes via membrane receptors. The well-characterized membrane receptor for bile-acids TGR5 was not expressed in these cells, whereas other G-protein-coupled receptors which play an important role in cardiac physiology, namely the muscarinic receptors, have been unexpectedly found to mediate the cardiac effects of bile acids. Here we unequivocally demonstrate that TC can bind to the muscarinic M_2_ receptor on the membranes of CHO-M2 cells and neonatal cardiomyocytes. TC serves as a partial agonist of this receptor. The partial agonism has been confirmed at the level of the second messenger cAMP and in several functional assays. Previous studies have known that in ventricular cardiomyocytes M_2_ muscarinic receptors inhibit cAMP production[36]. We show that TC induced a partial muscarinic effect at the cAMP level which was characterized by ∼30% inhibition of intracellular cAMP levels compared to carbachol (Fig. 3b,c). At the functional level, TC reduced the rate of contraction with slightly lower efficacies compared to carbachol, but the potency of TC in inhibiting contraction was much lower (Fig. 2a,b), supporting the idea of its partial agonistic effect. This effect was dependent on the G_i_-proteins and was blocked by the selective M_2_ receptor antagonist methoctramine and not by M_1_ and M_3_ receptor antagonists (Fig. 4c). It is known that stimulation of muscarinic M_2_ receptors within the mammalian heart[37], [38], modulates pacemaker activity and atrioventricular conduction, and directly (in atrium) or indirectly (in ventricles) influences the force of contraction[39]. Indeed, knocking down M_2_ receptors in the mouse abolishes bradycardia in response to carbachol[36], emphasizing the functional importance of this receptor subtype. Stimulation of the heart with the muscarinic receptor agonist carbachol causes a decrease in heart rate (i.e. bradycardia) in mice[36].

In addition, siRNA knockdown of the M_2_ receptor completely abolished the negative effect of TC on contraction (Fig. 4 and 5) and protected against the arrhythmogenic effect of TC which was characterized by a pronounced calcium desynchronization (Fig. 6). While it is generally assumed that parasympathetic stimulation of the heart acts through the M2-muscarinic acetylcholine receptors to regulate channel activity and subsequent cardiac inotropic and chronotopic status, there are varieties of proposed mechanisms. The intracellular Na^+^ current increases in guinea pig ventricles [40]and in sheep cardiac purkinje fibers [41] in the presence of either acetylcholine or carbachol. Alteration of Na^+^/K^+^ and Na^+^/Ca^2+^ pumps was also proposed to be the M2 agonist stimulant [41], [42,42]. More recently it has been shown that the (PTX)-sensitive Gα_i2_ coupling protein but not Gα_i3_ is required for the inhibitory action of muscarinic receptor on cell contractility and L-type calcium current in adult ventricular myocytes [43].

The structural basis for the specific and functional interaction of TC with muscarinic receptors is not fully understood, but it is possible to speculate that structural similarity exists between TC and other muscarinic receptor agonists (Fig. S6). Considerable likeness can be found between the portion of carbachol and TC side chain. It is electrostatic concordance between the two molecules, rather than perfect atomic alignment that may allow for the similar interaction with the muscarinic receptor. The ester carbonyl group of carbachol which carries a partial negative charge aligns with sulfonic acid in TC, and the amide of TC aligns with the ammonium ion of carbachol. It is likely that the interaction of these molecules with muscarinic receptors is based on electrostatic binding since two other molecules with similar interaction with muscarinic receptors, LCT and acetylcholine, share overall charge composition but not the atomic alignment **[26], [27]**.

In conclusion, we have identified partial agonism at the M_2_ receptor as a novel mechanism for bile acid-induced arrhythmia in a model of the fetal heart. Abolishing the M_2_ receptor resulted in elimination of the TC-induced-arrhythmia in cardiac tissue as revealed but the mechanical and optical mapping experiments respectively (Fig. 5, Fig. 6). The findings of this study indicate that muscarinic M_2_ receptor might be identified as a new therapeutic target to prevent fetal arrhythmia associated with the cholestasis of pregnancy and other diseases.

## Materials and Methods

### Reagents

^3^H-taurocholic acid (specific activity 5 Ci/mmol) and Ultima Gold scintillation cocktail were purchased from PerkinElmer (Boston, MA). All chemicals were purchased from Sigma-Aldrich (Gillingham, UK) unless otherwise stated.

### Neonatal rat Cardiomyocytes

Ventricular neonatal rat myocytes (NRCM) were isolated from the hearts of 1–2 day old Wistar rats according to Gorelik et. al, 2002[11] and under approval from the NHLI Imperial College London ethical committee (No. 1213456789). Experimental procedures using animals were performed in accordance with the U.K. Animals (Scientific Procedures) Act, 1986. Cells were kept in plating media (DMEM supplemented with 17% Medium 199 (v/v), 10% horse serum (v/v), 5% foetal calf serum (v/v), 200 µg/ml streptomycin, 200 U/ml penicillin). Cells were used following 2–3 days culture when small networks of cardiomyocytes had formed.

### ^3^H-Taurocholate (^3^HTC) Influx Assay

^3^H-taurocholic acid (specific activity 5 Ci/mmol) and Ultima Gold scintillation cocktail were purchased from PerkinElmer (Boston, MA). All chemicals were purchased from Sigma-Aldrich (Gillingham, UK) unless otherwise stated. NRCM and primary human hepatocytes (material and methods S1) seeded into 24-well plates were incubated at 4°C/37°C with 250 µl of influx media consisting of 0.2 µmol/L **^3^**HTC and 0–99.8 µmol/L TC in L-15 media for 0–60 minutes. Influx activity was stopped by washing with 500 µl of ice-cold 1 mmol/L TC and cells lysed with 250 µl of RIPA buffer (Sigma-Aldrich Gillingham, UK), 175 µl of which was mixed with 2 ml scintillation cocktail for radioactivity counting and the remainder used to assay protein quantity (Pierce BCA protein assay kit, Cramlington, UK) for normalisation. Cell membranes isolated from NRCM (20 µg protein) were incubated for 2 h at room temperature in assay buffer (25 mmol/L phosphate buffer saline, 5 mmol/L MgCl_2_, pH 7.4) with 0.1–10 nmol/L [3H]-N-methylscopolamine ([3H]-NMS) (Amersham, Freiburg, Germany). Nonspecific binding was determined in the presence of 10 µmol/L atropine and reactions terminated by vacuum filtration through GF/B glass fibre filters (Millipore, Schwalbach, Germany).

### Radioligand binding assays

Cell membranes isolated from NRCM (20 µg protein) were incubated for 2 h at room temperature in assay buffer (25 mmol/L phosphate buffer saline, 5 mmol/L MgCl_2_, pH 7.4) with 0.1-10 nM [3H]-N-methylscopolamine ([3H]-NMS) (Amersham, Freiburg, Germany). Nonspecific binding was determined in the presence of 10 µmol/L atropine and reactions terminated by vacuum filtration through GF/B glass fibre filters (Millipore, Schwalbach, Germany).

### RNA isolation and quantitative PCR (Q-PCR)

Total RNA was isolated from NRCM using the Qiagen RNEasy kit. 0.5 µg of RNA was reverse-transcribed using the Advantage RT-for-PCR Kit (Takara Biosciences, Saint-Germain-en-Laye, France). Relative gene expression was assayed using q-PCR detection of SYBR Green (JumpStart ReadyMix, Sigma-Aldrich), calculated using the ΔΔCt method and the following gene-transcript levels normalised to l19: *ntcp, shp, fxr (n1rh4)*and *mrp3* (for sequences Table S2).

### Nuclear protein extraction and western blot

NRCM and human hepatocytes cells line Huh7 seeded in 6-well plates were treated with 0.2 µmol/L TC for 0–60 minutes. Huh7 cells were a kind gift from Dr. Louise Collins The Rayne Institute, London. Nuclear protein was extracted with the NucBuster protein extraction kit (Novagen) according to manufacturer's protocol. Nuclear proteins were run on a 10% SDS-PAGE and analyzed by western blot. For detection of fxr protein, cells were lysed in ice-cold RIPA buffer and sonicated for 45 seconds on ice. Lysates were centrifuged at 10,000 rpm for 15 minutes at 4°C. Protein concentration was determined using the BCA assay kit (Perbio), 30 µg protein was fractionated on 10% SDS-PAGE gels and transferred to Protran nitrocellulose membrane (Schleicher and Schuell) by submerged transfer apparatus (Bio-Rad) in (25 mmol/L,Tris, 192 mmol/L glycine, 20% v/v methanol. Membranes were blocked for 1 hour at room temperature with 5% nonfat milk in PBS containing 0.05% Tween-20 and incubated with antibodies specific to fxr (1∶2000) or β-Actin (1∶10000; Santa Cruz Biotechnology) overnight at 4°C. The blots were probed with 1∶7000 dilution of horseradish peroxidase (HRP)-conjugated anti-mouse IgG and visualized using the ECL system (Amersham Biosciences). Blots were exposed to autoradiographic X-ray film and bands were quantified with ImageJ software and normalised again to β-Actin.

### Immunohistochemistry and Immunocytochemistry

Preparations were washed with PBS, fixed for 10 minutes with 4% (v/v) formaldehyde, washed with PBS and permeabilized for 40 minutes with 0.2% Triton X-100 (v/v). Post-permeabilisation, NRCM were blocked with 0.1% bovine serum albumin (w/v) for 15 minutes and subsequently probed overnight at 4°C with an anti-fxr antibody (1∶100; R&D Systems). Following antibody incubation, NRCM were incubated with fluorescein-conjugated secondary antibody for 1 hour at RT. NRCM nuclei were stained with DAPI for 20 minutes at 37°C. The cells were washed with PBS and the coverslips were mounted on glass slides using Vectashield.

For immunofluorescent studies, isolated **rat** Kupffer cells and **neonatal** cardiomyocytes were seeded on glass coverslips and fixed in 100% methanol (5 min, −20°C). Cryosections (5 µm) of perfused rat liver were prepared with a Leica cryotome, air-dried for 1 hour and fixed in 100% methanol (5 min, −20°C). An antibody against the C-terminus (aa 306–329) of rat TGR5 was used at 1∶150[25]. A Cyanin-3 conjugated secondary antibody (Jackson Immuno Research Laboratories, West Grove, PA) was diluted 1∶500 and Hoechst 34580 (Invitrogen) was added at 1∶10,000 respectively. Immunostained samples were analyzed on Zeiss LSM 510 META confocal microscope (Zeiss, Oberkochen, Germany) using the same settings for all samples.

### Assessment of beating frequencies

Beating frequencies in each cell were visually recorded according to Clark *et. al*. (1991)[44]. Cardiomyocytes were pre-incubated at 37°C humidified with 5% CO_2_ with 1 µmol/L atropine, 1 µmol/L methoctramine, 1 µmol/L pirenzepine, 1 µmol/L 4-DAMP, 100 µmol/L carbachol (CCh), 50 nmol/L Pertussin Toxin (PTX), 100 µmol/L oleanolic acid (0.1 mmol/L) or 10 µM GW4064 for 30 minutes pre-bile acid treatment. NRCM contraction rates were counted under a light microscope. NRCM were treated with 0.2/1 mmol/L TC for 10 minutes and contraction rate re-counted.

### siRNA-mediated Gene Silencing of chrm2

siRNA against M_2_ and control scramble sequences were purchased from Thermo Scientific Dharmacon (siM_2_ catalogue M-092972, siScramble catalogue D-001210). 100 nmol/L concentration of siM_2_/Scramble was transfected into 60–70% confluent NRCM using Dharmafect1 for 48 hours according to manufacturer's protocol. Post-transfection, NRCM were washed and cultured in phenol red free DMEM with 2% DCC-FCS. M_2_ knockdown levels were assessed with qRT-PCR.

### Measurement of contraction using Scanning Ion Conductance Microscope (SICM)

The scanning ion conductance microscope (SICM) provides accurate measurement of the rate, rhythm and amplitude of contraction on NRCM. The physiological L-15 medium (Gibco, Parsley, UK) was used as the bath and micropipette solution.

### Optical recording of Calcium transients

The characteristics of Ca^2+^ transients were assessed optically after staining the preparations with 5 µg/ml of Fluo-4 AM (Invitrogen) for 20 min at 37°C. During the experiments, preparations were continually superfused at 36°C with Hanks' balanced salt solution (HBSS) containing (mmol/L) NaCl 137, KCl 5.4, CaCl_2_ 1.3, MgSO_4_ 0.8, NaHCO_3_ 4.2, KH_2_PO_4_ 0.5, NaH_2_PO_4_ 0.3 and HEPES 10. The solution was titrated to pH 7.40 with 1 mol/L NaOH. Changes in fluorescence corresponding to intracellular Ca^2+^ changes were assessed using a fast CMOS camera (MiCAM Ultima, Scimedia) coupled to a Nikon inverted microscope Ti/U equipped for epifluorescence (magnification 20×, N.A 0.75). This system is able to record calcium activity in an area measuring 1 mm×1 mm with a spatial resolution of 5 µm and a temporal resolution of 1 ms. The assessment of calcium transient in a given preparation was limited to 2-5 recordings, 4 s each, in order to avoid phototoxic effects of the dyes. Optical raw data were analyzed using dedicated software from the camera manufacturer (BVAnalyze V8.02, SciMedia). After offset correction the maximum dF/F was setting at 10% in order to scale the traces with the Ca^2+^ amplitude measured in pre-treated preparations. Experiments were performed with 3-day-old preparations.

### Acute effect of Taurocholate on Calcium Transient

After measuring Ca^2+^transients in normal superfusion solution (HBSS), the same preparations (control, scramble and siRNA M2) were superfused with an HBSS solution containing TC 0.2 mmol/L and Ca^2+^ transients were recorded again after 20 min perfusion in the same conditions. Cardiomyocyte clusters were included in the analysis if they fulfilled the following criteria: (i) the clusters had to denote spontaneous beating activity, (ii) the clusters had to consist only of cardiomyocytes and (iii) the clusters had to be isolated with a maximum dimension of 100×100 µm.

### FRET imaging of cAMP

FRET imaging of cAMP in living neonatal cardiomyocytes was performed in cells infected for 48 h with Epac2-camps adenovirus. Cells were washed twice and measured at room temperature in buffer A. The imaging system was built around a Nikon TE2000 microscope equipped with a mercury lamp (HB0103W/2, Osram), EX436/20 excitation filter combined with DM455 dichroic mirror. Cell fluorescence was split into YFP and CFP channels using a DualView (Optical Insights) equipped with 535/40 and 480/30 emission filters and monitored by the ORCA-ER CCD camera (Hamamatsu, Welwyn Garden City, UK). FRET ratios were corrected for the bleedthrough of CFP into the YFP channel and analyzed using Origin software (OriginLab Corporation, Northampton, MA).

## Supporting Information

Material and Methods S1 Cell culture of Primary Human Hepatocytes. Human liver tissue was taken at the tumor-free margins of resection specimens removed by surgical intervention for secondary liver tumors, with fully informed consent and local research ethics approval (RFH 38-2000).
(0.03 MB DOC)

Table S1 Primers for gene expression.
(0.03 MB DOC)

Table S2 Fold decrease in the expression of bile acid transporters and receptors in rat adult heart and fetal heart as compared to adult rat liver. Quantitative RT-PCR was performed on Applied Biosystems Prism on RNA extracts of respective tissues. First we looked at the expression of various transporters that have been previously reported to be involved in bile acid transport in other cells, mostly hepatocytes. As a control, bile acid transporter expression in the liver was examined. We wanted to compare the levels of expression between adult heart and fetal heart, as these differences may contribute to the differences in susceptibility of the fetal heart to bile acids. We also wanted to know whether cultures neonatal cardiomyocytes that we use as a model of fetal heart behave similarly to fetal heart in respect of transporter expression. Table shows the expression of bile acids and nuclear transporters in neonatal heart and cardiomyocytes as compared to adult liver. Using qRT-PCR we have shown that of all the main transporters mdr2, ntcp2, shp and fxr are expressed in adult and fetal rat hearts as well as in rat neonatal cardiomyocytes cultures compared to adult rat liver. The expression of mrp2 is significantly lower. All the genes studied are expressed substantially less in the adult and fetal hearts and the cultured cardiomyocytes than in the adult liver. Most genes show similar level of gene expression between adult heart, fetal heart and cultured cardiomyocytes. There is no significant difference between adult and fetal heart.
(0.03 MB DOC)

Figure S1 Specific binding of TC to the CHO-M2 cell membranes measured as described in Methods. A representative competition displacement curve for TC is shown.
(0.41 MB TIF)

Figure S2 The muscarinic receptor is involved in CCh-induced arrhythmia. Contraction of NRCM expressed as beat per minutes (bpm).Scramble (non-targeting) siRNA and M2 siRNA knockdown of cells. (* Control vs P<0.001); n≥3 observations).
(0.41 MB TIF)

Figure S3 Efficiency of the siRNA knockdown is confirmed by qRT-PCR. (* Control vs P<0.001); n = 3 observations.
(0.31 MB TIF)

Figure S4 Representative measurement of the amplitude contraction of NRCM using SICM. Scramble (non-targeting) siRNA cardiomyocytes showed regular contraction and TC- treatment with showed effect on rhythm and amplitude.
(0.34 MB TIF)

Figure S5 Representative measurement of the amplitude contraction of rat neonatal cardiomyocytes using SICM. siRNA M2 knockdown NRCM showed regular contraction and TC treatment showed no effect on rhythm of contraction.
(0.36 MB TIF)

Figure S6 Chemical structures of Taurocholate (TC) and Carbachol (CCh).
(0.38 MB TIF)

Video S1 Representative video of intracellular Calcium dynamics recorded in NRCM pre and post-treatment with 0.2 mmol/L TC.
(2.29 MB WMV)
