# Supplementary material for: Bile Acid-Induced Arrhythmia Is Mediated by Muscarinic M2 Receptors in Neonatal Rat Cardiomyocytes
Source: PLoS One. 2010 Mar 15;5(3):e9689. doi: 10.1371/journal.pone.0009689 (PMC2837738; doi:10.1371/journal.pone.0009689)
Supplement: Material and Methods S1 — Cell culture of Primary Human Hepatocytes. Human liver tissue was taken at the tumor-free margins of resection specimens removed by surgical intervention for secondary liver tumors, with fully informed consent and local research ethics approval (RFH 38-2000). (0.03 MB DOC) [file pone.0009689.s001.doc]

Supplementary material and methods related to the manuscript:

**Bile acid-induced arrhythmia is mediated by muscarinic M_2_ receptors in the model of fetal heart.**

**Material and Methods**

***Cell culture of Primary Human Hepatocytes.*** Human liver tissue was taken at the tumor-free margins of resection specimens removed by surgical intervention for secondary liver tumors, with fully informed consent and local research ethics approval (RFH 38-2000). PHH were isolated by collagenase perfusion as described in [1].

Reference

Reference List

1. Selden C (2007) Growth factors improve gene expression after lentiviral transduction in human adult and fetal hepatocytes. Journal of Gene Medicine 9: 67.
