# Supplementary material for: Bile Acid-Induced Arrhythmia Is Mediated by Muscarinic M2 Receptors in Neonatal Rat Cardiomyocytes
Source: PLoS One. 2010 Mar 15;5(3):e9689. doi: 10.1371/journal.pone.0009689 (PMC2837738; doi:10.1371/journal.pone.0009689)
Supplement: Table S1 — Primers for gene expression. (0.03 MB DOC) [file pone.0009689.s002.doc]

Table S1. Primers for gene expression

| Gene | Forward primer | Reverse Primer |
| --- | --- | --- |
| *l19* | ATCTCAAGCAAACACCATTCC | TTGATGATGCTGTCTAGTACC |
| *chrm2* | TTGTGGCTGGATCCCTCAGT | TGGCGGTTGACTTTAATGGAA |
| *fxr* | TGACAAAGAAGCCGCGAA | CACACAGCTCATCCCCTTTTATT |
| *shp* | CCTTGGCTAGCTGGGTACCA | GTCCCAAGGAGTACGCATCTT |
| *ntcp* | ATCTCAAGCAAACACCATTCC | GATCATGCCTCCCTTAAGGA |
| *mdr2* | GAAGGAATGTGGCCGGGATAA | GGCCCGGGTGGGATAAT |
| *mrp2* | CTGGAGCTGGTTGGAAACTTG | CGTCCCCGGTTAAGGTTTTT |
