# Supplementary material for: Bile Acid-Induced Arrhythmia Is Mediated by Muscarinic M2 Receptors in Neonatal Rat Cardiomyocytes
Source: PLoS One. 2010 Mar 15;5(3):e9689. doi: 10.1371/journal.pone.0009689 (PMC2837738; doi:10.1371/journal.pone.0009689)
Supplement: Table S2 — Fold decrease in the expression of bile acid transporters and receptors in rat adult heart and fetal heart as compared to adult rat liver. Quantitative RT-PCR was performed on Applied Biosystems Prism on RNA extracts of respective tissues. First we looked at the expression of various transporters that have been previously reported to be involved in bile acid transport in other cells, mostly hepatocytes. As a control, bile acid transporter expression in the liver was examined. We wanted to compare the levels of expression between adult heart and fetal heart, as these differences may contribute to the differences in susceptibility of the fetal heart to bile acids. We also wanted to know whether cultures neonatal cardiomyocytes that we use as a model of fetal heart behave similarly to fetal heart in respect of transporter expression. Table shows the expression of bile acids and nuclear transporters in neonatal heart and cardiomyocytes as compared to adult liver. Using qRT-PCR we have shown that of all the main transporters mdr2, ntcp2, shp and fxr are expressed in adult and fetal rat hearts as well as in rat neonatal cardiomyocytes cultures compared to adult rat liver. The expression of mrp2 is significantly lower. All the genes studied are expressed substantially less in the adult and fetal hearts and the cultured cardiomyocytes than in the adult liver. Most genes show similar level of gene expression between adult heart, fetal heart and cultured cardiomyocytes. There is no significant difference between adult and fetal heart. (0.03 MB DOC) [file pone.0009689.s003.doc]

Supplementary Table 2.

| Gene | adult rat heart | fetal rat heart | rat neonatal cardiomyocytes |
| --- | --- | --- | --- |
| *mdr2* | 2 | 3 | 2 |
| *mrp2* | 22 | 20 | 29 |
| *ntcp* | 3 | 4 | 4 |
| *shp* | 1.5 | 2 | 2 |
| *fxr* | 5 | 3 | 4 |
