# Supplementary figures and images for: Bile Acid-Induced Arrhythmia Is Mediated by Muscarinic M2 Receptors in Neonatal Rat Cardiomyocytes
Source: PLoS One. 2010 Mar 15;5(3):e9689. doi: 10.1371/journal.pone.0009689 (PMC2837738; doi:10.1371/journal.pone.0009689)

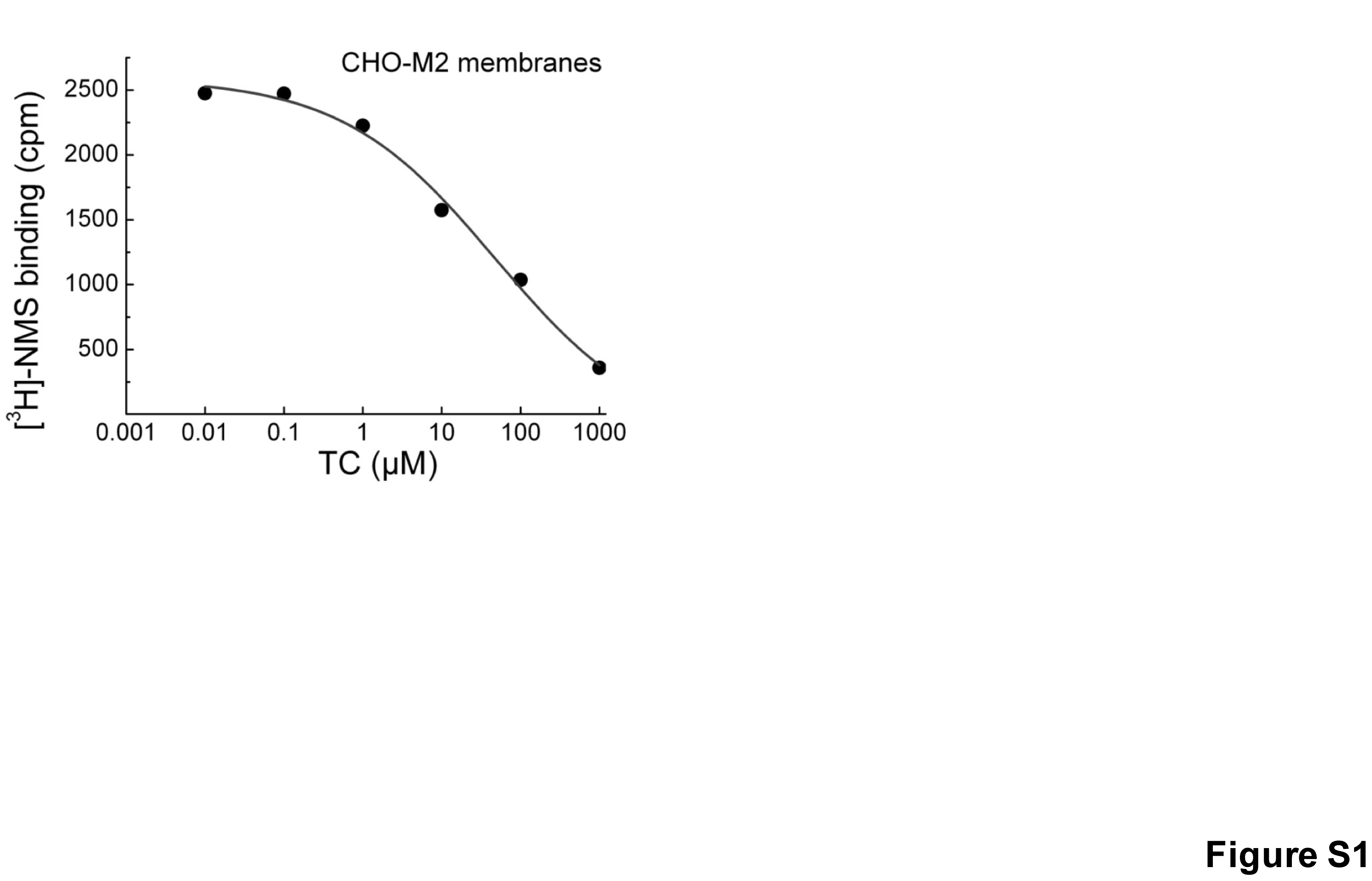

Supplement: Figure S1 — Specific binding of TC to the CHO-M2 cell membranes measured as described in Methods. A representative competition displacement curve for TC is shown. (0.41 MB TIF) [file pone.0009689.s004.tif]

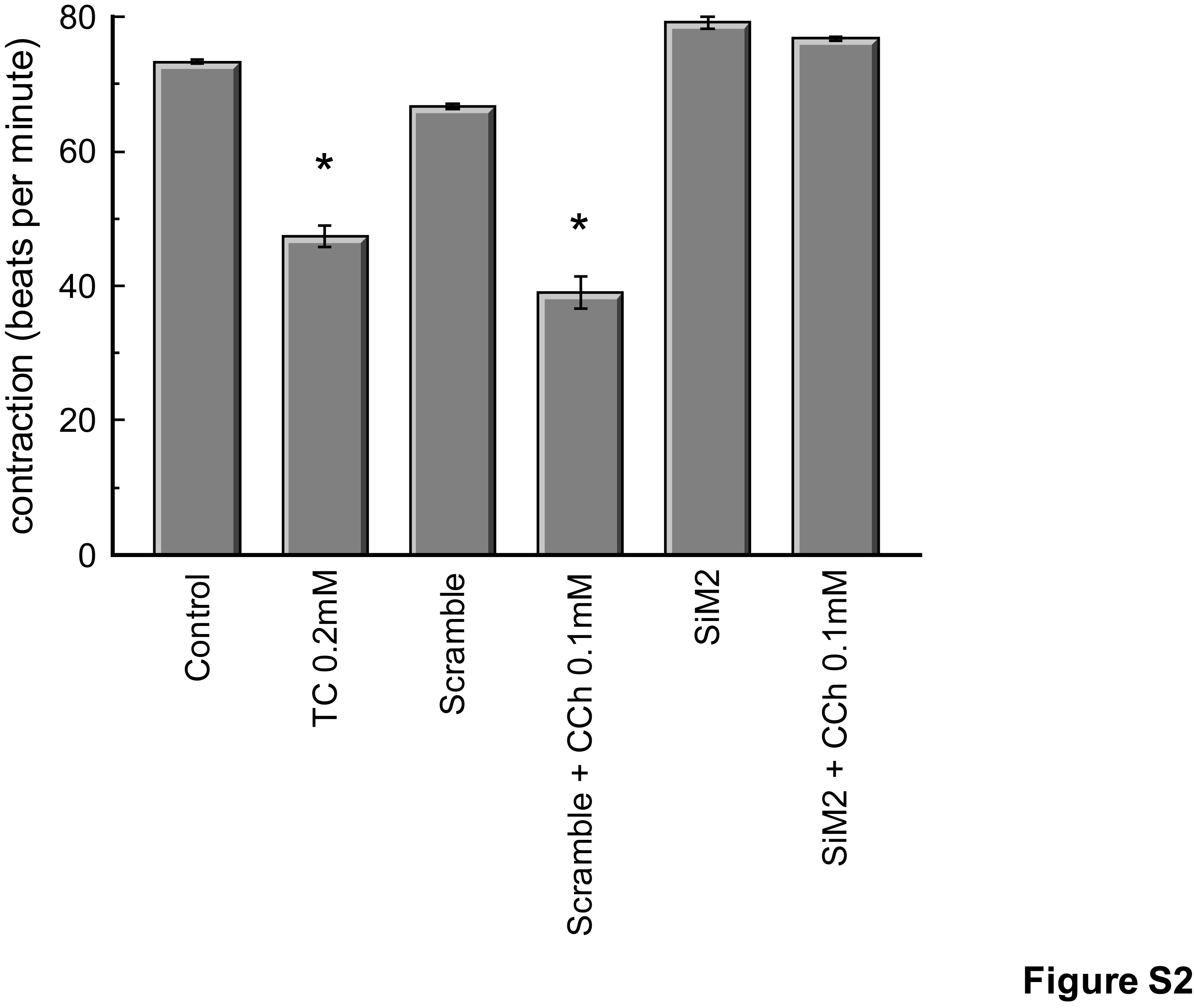

Supplement: Figure S2 — The muscarinic receptor is involved in CCh-induced arrhythmia. Contraction of NRCM expressed as beat per minutes (bpm).Scramble (non-targeting) siRNA and M2 siRNA knockdown of cells. (* Control vs P<0.001); n≥3 observations). (0.41 MB TIF) [file pone.0009689.s005.tif]

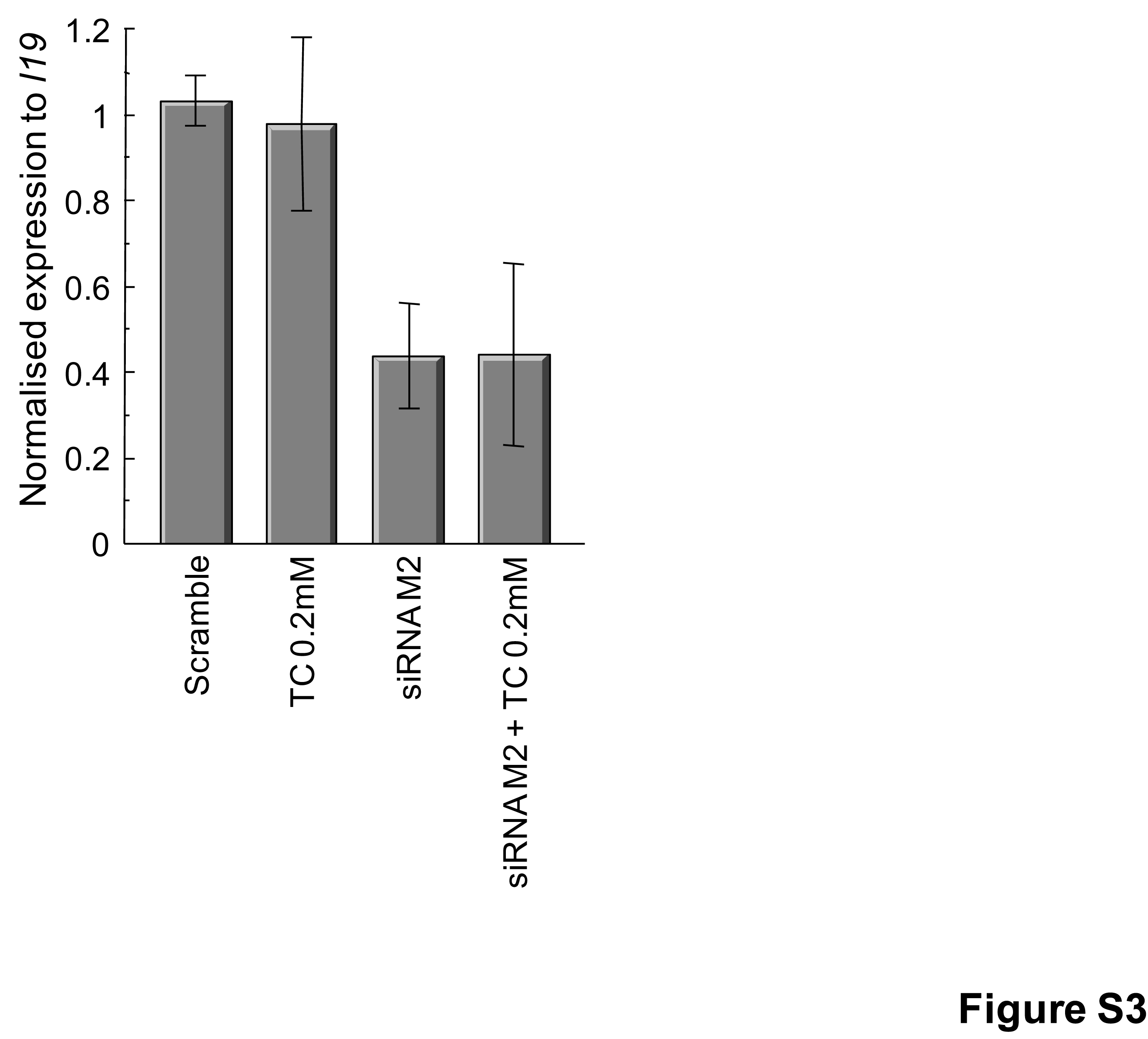

Supplement: Figure S3 — Efficiency of the siRNA knockdown is confirmed by qRT-PCR. (* Control vs P<0.001); n = 3 observations. (0.31 MB TIF) [file pone.0009689.s006.tif]

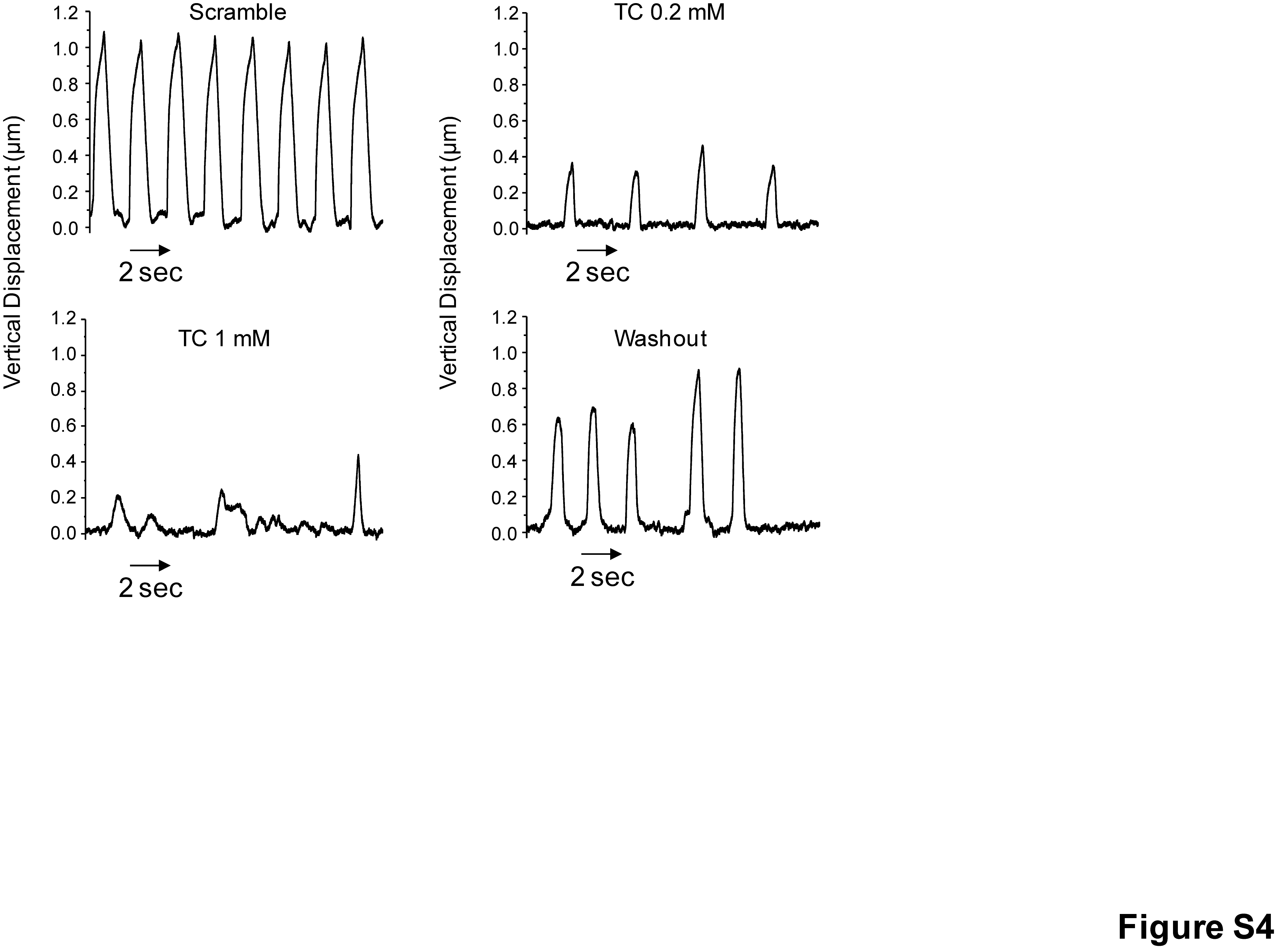

Supplement: Figure S4 — Representative measurement of the amplitude contraction of NRCM using SICM. Scramble (non-targeting) siRNA cardiomyocytes showed regular contraction and TC- treatment with showed effect on rhythm and amplitude. (0.34 MB TIF) [file pone.0009689.s007.tif]

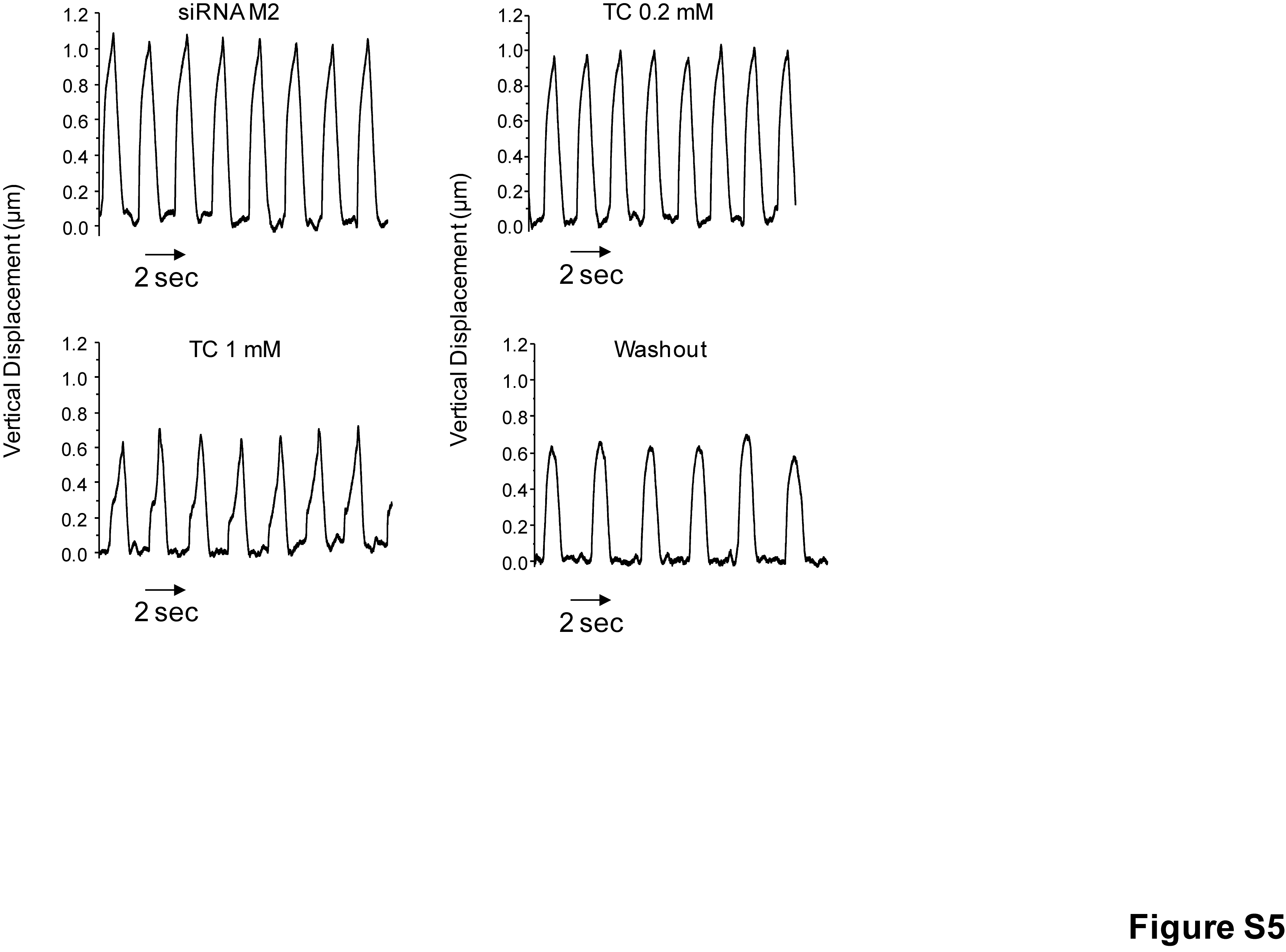

Supplement: Figure S5 — Representative measurement of the amplitude contraction of rat neonatal cardiomyocytes using SICM. siRNA M2 knockdown NRCM showed regular contraction and TC treatment showed no effect on rhythm of contraction. (0.36 MB TIF) [file pone.0009689.s008.tif]

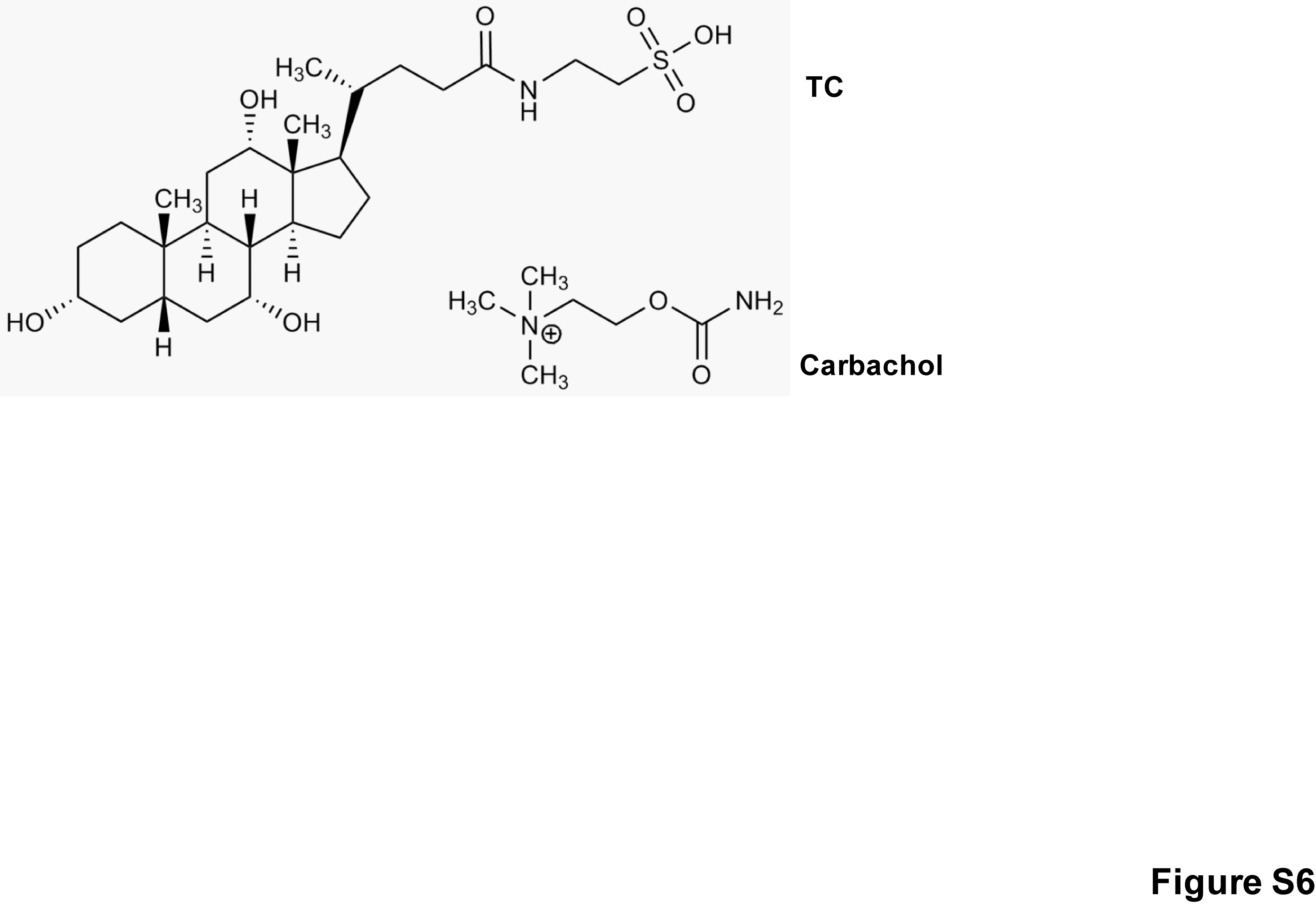

Supplement: Figure S6 — Chemical structures of Taurocholate (TC) and Carbachol (CCh). (0.38 MB TIF) [file pone.0009689.s009.tif]
